# Traditional Uyghur medicine *Quercus infectoria* galls water extract triggers apoptosis and autophagic cell death in colorectal cancer cells

**DOI:** 10.1186/s12906-020-03167-0

**Published:** 2020-12-03

**Authors:** Hui Zhang, Yongbing Wang, Jiayang Liu, Kudelaidi Kuerban, Jian Li, Mubarak Iminjan, Li Ye

**Affiliations:** 1 Department of Biological Medicines & Shanghai Engineering Research Center of Immunotherapeutics, School of Pharmacy, Fudan University, Shanghai, 201203 China; 2 Pudong New Area People’s Hospital Affiliated to Shanghai University of Medicine and Health Sciences, Shanghai, 201200 China; 3 Endoscopy Center, Minhang Branch of Zhongshan Hospital, Fudan University, Shanghai, 200433 China; 4 Department of pharmaceutical and physical chemistry, College of pharmacy, Xinjiang Medical University, Xinjiang, 830011 China

**Keywords:** Quercuse infectoria galls, Colorectal cancer, Apoptosis, Autophagy, Reactive oxygen species

## Abstract

**Background:**

The water extract of Quercuse infectoria galls (QIG) is the active ingredient of Uyghur medicine Xipayi Kui Jie’an (KJA) which has promising therapeutic effects on Ulcerative Colitis (UC) as an alternative medicine. Considering the relationship between UC and the development of colorectal cancer (CRC), the present work aims to explore the direct anti-CRC activity of QIG extract.

**Methods:**

CCK8 assay and flow cytometry were used to detect cytotoxicity and apoptosis. Transmission electron microscopy (TEM), flow cytometry, laser confocal and western blotting were performed to examine autophagy. We also adopted Reactive Oxygen Assay kit, as well as transwell and wound healing tests to study the underlying mechanism of QIG against CRC cells.

**Results:**

First, we found that QIG extract could suppress the viability of CRC cells and trigger caspases-dependent apoptosis. Subsequently, we proved for the first time that QIG extract also triggered autophagic cell death in CRC cells, which together with apoptosis contributed to the cytotoxic effect on CRC cells. Further investigation revealed that QIG-induced cytotoxicity associated with intracellular ROS accumulation which could suppress the AKT/mTOR signaling pathway, and then induce autophagy and inhibit cell growth. Besides, Erk signaling pathway was also involved in the process of autophagic cell death. Moreover, QIG extract also influenced EMT process and inhibited CRC cell migration.

**Conclusion:**

Altogether, this study provides a basis for the utilization of QIG as an alternative medicine for CRC prevention and treatment.

## Introduction

Global cancer statistics data show the prevalence of colorectal cancer (CRC) ranks third in the world in 2018, with approximately 1,800,000 people being diagnosed with CRC each year [1]. Despite current advances in immunotherapy and targeted therapy, the prognosis is poor and the 5-year survival rate remains unacceptably low especially for patients with advanced CRC [2]. Many combination therapy strategies comprising natural compounds have been explored to sensitize CRC cells to conventional cytotoxic therapy as well as immune or targeted therapy [3]. In addition, many of natural products are mutitargeted agents inducing CRC cells’ apoptosis, inhibiting tumor growth and proliferation or initiating cell cycle arrest [4].

The famous Uyghur medicine Xipayi Kui Jie’an (KJA), as an alternative medicine developed from the “Xipayi gingiva protective solution”, has potential therapeutic effects on Ulcerative Colitis (UC) [5]. KJA contains the water extract of Quercuse infectoria galls (QIG), an insect gall produced by *Cynips gallae tinctoriae* wasp on the tree branches of *Quercus infectoria Oliv,* mainly distributes in Syria Greece, Iran and Asia Minor [6]. QIG is rich in tannins with about 50–70%, followed by gallic acid, ellagic acid, hexamethyl, syringic acid, amentoflavone, sitosterol and glucose propionic acid [7, 8]. It has various pharmacological activities including antifungal, antiviral, insecticidal, astringent, wound healing, gastric protective effects and antiulcer, and the most important use of it is to treat UC [9]. Besides, its main constituent gallic acid displays inhibitory effect on tumor cells through regulating multiple signaling pathways involved in carcinogenesis [10]. UC is characterized by chronic inflammation and ulceration in the digestive tract and patients with UC were at high risk of CRC [11]. Besides genetic and environmental factors, inflammation is generally considered as a critical factor leading to the development of CRC [12]. Considering the therapeutic effects of KJA on UC and the relationship between UC and CRC, we wonder whether the water extract of QIG has direct anti-tumor activity to CRC cells.

Apoptosis and autophagy are two distinctive kinds of programmed cell death (PCD) which together determine the fate of tumor cells. While the initiation of apoptosis definitely results in cell death, the influence of autophagy is complex and may be pro-survival or commit suicide [13]. Generally, autophagy plays a protective role and contributes to drug resistance during chemotherapy [14, 15]. It was reported that natural anti-tumor drug paclitaxel could trigger autophagy and apoptosis simultaneously in cervical cancer cells, and suppression of the autophagy could enhance the therapeutic effect of drug [16]. However, some tumor therapeutic drugs and approaches can also induce autophagic cell death (ACD) which ultimately enhances the apoptosis and cell growth suppression [17, 18].

The present work aims to explore whether QIG can induce apoptosis and autophagy in CRC cells, as well as their roles in cell fate. We found that both apoptosis and autophagy were triggered by QIG, which together contributed to the cytotoxic effect on CRC cells. In addition, the signaling pathways related to QIG-induced cytotoxicity were investigated. We hope our results can provide experimental basis for further in vivo experiments and the application of QIG in CRC therapy as a complement and alternative medicine.

## Materials and methods

### Preparation of the QIG aqueous extract

The water extract of QIG was used in this study. The air-dried materials of QIG (20190303) were purchased from Xinjiang Autonomous Region Traditional Uyghur Medicine Hospital (Urumqi, China). The samples were ground and then mixed with distilled water for 1 h at a volume ratio of 1: 8. The aqueous extract was boiled 3 times for 30 min each, and then filtered and concentrated under reduced pressure. Finally, the water extract was evaporated to dryness in a water bath, then passed through an 80 mesh screen. The extract was examined and standardized by TLC and HPLC according to current Chinese Pharmacopoeia. QIG extract was diluted with PBS and filtered with a 0.22 μm filter before each used for cell experiments.

### Cell lines and treatment

HT-29 (CRC human cell line) and CT-26 (CRC murine cell line) were purchased from Cell Bank of Chinese Academy of Sciences (Shanghai, China) and the passage number of them in this experiment was between 4 and 5. Cell medium was RPMI-1640 or DMEM (CORNING) containing 100 IU/ml penicillin (Beyotime Biotechnology) and 10% fetal bovine serum (Gibco). The cell incubator maintained 5% CO_2_ and 37 °C.

### Materials and antibodies

Antibodies against LC3, Beclin-1, p62, Cleaved-PARP, Cytochrome c, Bax, Bcl-2, ERK/pERK1/2, caspase 9, p-p70S6K, mTOR/p-mTOR, E-cadherin, p-4EBP1, caspase 3, EpCAM, N-cadherin, GAPDH, COX IV and β-Tubulin were purchased from Cell Signaling Technology (Danvers, MA, USA). BCA Protein Quantitation Kit and Annexin V-FITC/PI Apoptosis Detection Kit were obtained from KeyGen Biotech (Nanjing, China). Other reagent purchase information were as follows: Cyto-ID® Autophagy Detection Kit (Enzo Life Sciences, Farmingdale, NY, USA), Cell Counting Kit-8 (Meilun Biotechnology, Dalian, China), ROS Assay Kit (Beyotime Biotechnology, Haimen, China), Acridine Orange (Absin Bioscience, Shanghai, China).

### CCK8 assay

CRC cells (5 × 10^5^/ml) were added to a 96-well plate with 100 ul of cell suspension per well. After treating the cells with QIG at different concentrations (0.0125–0.5 mg/ml) for a certain time, CCK-8 reagent was added with 10 μl each pore. Then the mixture was incubated in a 37 °C incubator for 1.5 h, and the absorbance was tested with a microplate reader at 450 nm.

### Morphological analysis

CRC cells were administrated with QIG at 0.3 mg/ml or 0.5 mg/ml for 24 h. Cell morphology was detected by an inverted microscope (Nikon, Japan).

### Apoptosis assay

Cells were digested with 0.25% trypsin after treatment with 0 mg/ml, 0.1 mg/ml, 0.3 mg/ml and 0.5 mg/ml of QIG for 24 h, and then collected by centrifugation at 1500 r/min. After that, they were washed twice with PBS and dyed with Annexin V-FITC and PI for 20 min under 4 °C without light. The level of apoptosis was assessed by flow cytometry (Becton-Dickinson, NJ, USA).

### Western blotting test

HT29 cells were treated with RIPA Cell Lysis Buffer or Cytoplasmic and Mitochondrial Protein Extraction Kit (Sangon Biotech, China). BCA quantification kit was used to measure the total protein concentration. Next, an equal amount of protein (15 μg) was separated by SDS-PAGE electrophoresis and transferred to a polyvinylidene fluoride (PVDF) membranes. Then the PVDF membrane was blocked with 3% bovine serum albumin (BSA) for 2.5 h and incubated with primary antibody for 6 h at 4 °C. Finally, it was probed with peroxidase-conjugated secondary antibody and washed with TBST. ECL chemiluminescence substrate (Pierce, Rockford, IL, USA) was used for the immunoblot detection through ChemiDoc software (Bio-Rad, USA).

### Transmission electron microscopy

After incubation with 0.3 mg/ml of QIG for 1 day, HT-29 cells were collected and treated with pre-chilled glutaraldehyde. JEM 1410 transmission electron microscope was used to test the cell sections at 80 Kv (JEOL, Inc., USA).

### Confocal immunofluorescence analysis

HT-29 cells were seeded in 6-well glass dishes and treated with 0.3 mg/ml QIG for 24 h. Autophagy inducer rapamycin (50 nM) was used as a positive control and it was incubated with cells for 6 h. Thereafter, cells were washed with serum-free medium and treated with nuclear dye Hoechst 33342 and autophagy detection kit Cyto-ID® Green Dye for 20 min. Analysis was performed immediately in dark by a fluorescence microscope.

### Detection of acidic vesicular organelles (AVOs)

HT-29 cells were prepared in 6-well micro-plate and treated with 0 mg/ml, 0.1 mg/ml, 0.3 mg/ml and 0.5 mg/ml of QIG for 24 h respectively. Thereafter, cells were washed with PBS and stained with 5 μg/ml of Acridine Orange (AO) for 15 min at 37 °C. AO was a kind of weak fluorescent base, which could combine with AVOs to emit bright red fluorescence (650 nm), and combine with cytoplasm and nucleus to emit green fluorescence (515–545 nm) [19]. The AVOs images were observed by inverted fluorescence microscope at 40× objective. And the percentage of AOVs accumulation in HT-29 cells was measured by flow cytometry after cells were washed twice and resuspended with PBS.

### Measurement of intracellular ROS

Intracellular ROS generation was detected in HT-29 cells by Reactive Oxygen Species Assay Kit. DCFH-DA was non-fluorescent but can be oxidized to DCF with fluorescent by intracellular ROS. Cells were treated with QIG at different concentrations (0.05 mg/ml-0.5 mg/ml) for 24 h, and then co-incubated with DCFH-DA for 20 min. After washing cells with fresh culture medium without serum for three times, the fluorescence intensity of DCF was quantified by Tecan Infinite® 200 PRO microplate reader at 525 nm for emission wavelength and 488 nm for excitation wavelength.

### SiRNA transfection assay

Non-specific scrambled siRNA (siNO581512211471–10) and small-interfering siRNA ATG5–1 (siB08530151718), siRNA ATG5–2 (siG10726164423) and siRNA ATG5–3 (siB1273133305) were obtained from Guangzhou RiboBio Co. It was added in 6-well plates and delivered with Lipofectamine 3000 Transfection Reagent (Thermo Scientific, China) when the cell density reached 70–90%.

### Wound-healing assay

When the HT-29 cells were cultured into confluent monolayers in a six-well cell culture cluster, a 10-μl sterile tip was used to draw multiple lines on the cell layer and different concentrations of QIG were added to cells. The average level of cell migration was analyzed after obtaining an image at 0 h, 12 h and 24 h with an 10× objective lens of an inverted microscope.

### Transwell migration experiment

Experiments were implemented in 12-well cell culture plate (Corning), with 600-μl medium including 20% FBS on the lower compartments and 150-μl cells suspension on the upper chambers. QIG was co-incubated with cells at different concentrations. After 24 h, cells on the chambers were treated with 800-μl paraformaldehyde for 15 min and then dyed with crystal violet for 20 min. The migrated cells were identified using an 100x optical microscope after washing several times with PBS.

### Statistical analysis

Data of this work was showed as means ± standard deviations (SD). The statistical significance between groups was performed with GraphPad Prism 7 using two-tailed Student’s t test or one-way ANOVAs. It was regarded as statistically significant by *P* values < 0.05.

## Results

### Cytotoxicity induced by QIG on CRC cells

**Fig. 1:**
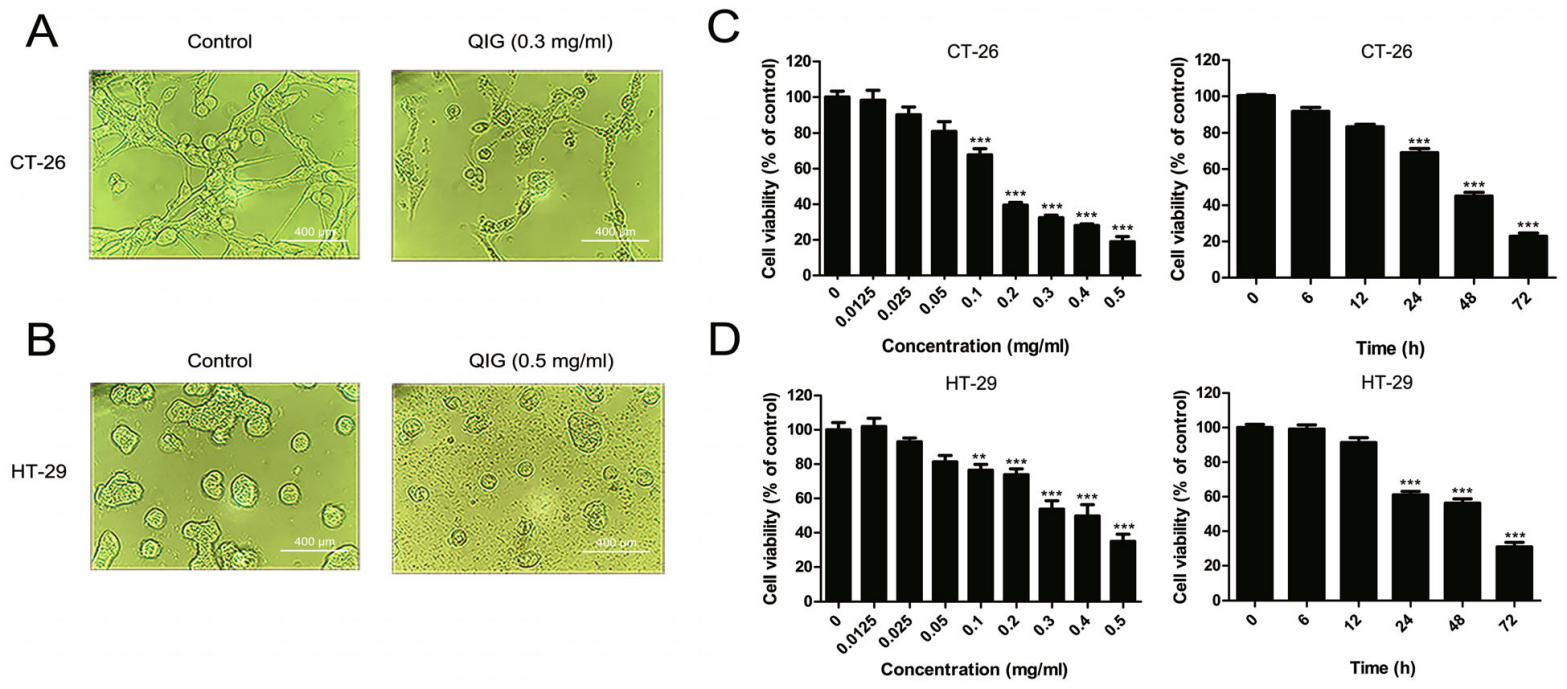
Cytotoxic effect of QIG on CRC cells. (**a**-**b**) CT-26 and HT29 cells were treated with 0.3 mg/ml or 0.5 mg/ml QIG for 24 h, and then the morphological alteration of cells was observed under microscope. (**c**-**d**) After treatment with QIG in different concentrations and times, the cell viability of CT-26 and HT29 cells was detected by CCK8 assay (IC50 of QIG on CT-26 = 0.1645 mg/ml, IC50 of QIG on HT-29 = 0.3655 mg/ml). The data were presented as mean ± SD (**P < 0.01, ***P < 0.001)

The growth and morphology of the CRC cells were photographed under an inverted microscope. After treated with 0.3 mg/ml or 0.5 mg/ml QIG for 24 h, the number of CT-26 and HT-29 cells decreased, along with cell shrinkage and the appearance of cell debris (Fig. 1a and b). In addition, the cytotoxicity of QIG was assessed by the CCK8 kit, and the data indicated that QIG suppressed the viability of CRC cells in a time and dose-dependent fashion (Fig. 1c and d). The IC_50_ of CT-26 cells was 0.1645 mg/ml after co-incubated with QIG for 24 h, and that of HT-29 was 0.3566 mg/ml.

### Caspase-dependent apoptosis was triggered by QIG in CRC cells

**Fig. 2:**
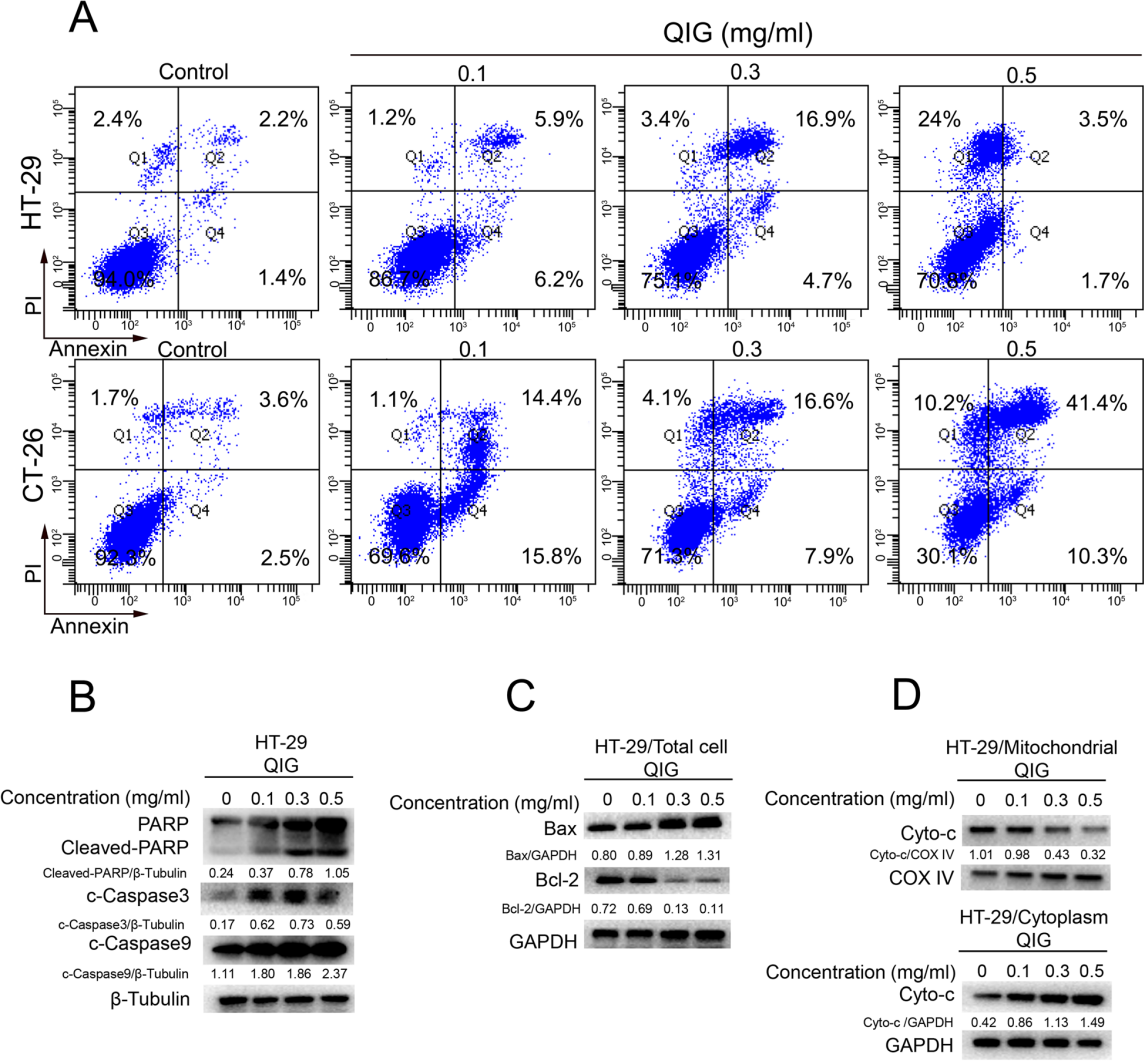
QIG induced caspase-dependent apoptosis in CRC cells. **a** After treated with 0.1 mg/ml, 0.3 mg/ml and 0.5 mg/ml of QIG for 24 h, HT-29 and CT-26 cells were stained with Annexin V/PI and the apoptosis was analyzed by flow cytometry. **b**-**c** HT-29 cells were dose-dependently treated with QIG, then the protein levels of PARP, cleaved-PARP, cleaved-caspase 3, cleaved-caspase 9, Bax, Bcl-2, β-Tubulin and GAPDH were detected by western blotting and quantified by Image J software. **d** The expression of Cyto-c in mitochondrial and cytoplasm were detected by western blotting and quantified by Image J software

In order to define whether QIG suppresses cell viability by inducing apoptosis, HT-29 cells and CT-26 cells were administrated with QIG for 24 h and dyed with Annexin V/PI. The apoptotic effect of QIG was assessed by flow cytometry. Comparing with the control group, the apoptosis ratio of HT-29 cells treated with QIG (0.3 mg/ml) significantly improved from 3.6 to 21.6%, and that of CT-26 cells was increased by 18.4% (Fig. 2a).

At the same time, we examined the influence of QIG on the levels of apoptosis-related proteins. It is well known that caspases families are cleaved and activated in the process of apoptosis [20]. In this study, western blotting test indicated the increased cleavage of PARP, caspase-3, caspase-9 after administered with QIG in HT-29 cells (Fig. 2b). In addition, we found that QIG treatment reduced the expression of anti-apoptosis protein Bcl-2, while increased that of pro-apoptosis protein Bax in HT-29 cells (Fig. 2c). Moreover, the down-regulation of Cyto-c in the mitochondria and up-regulation of Cyto-c in the cytoplasm indicated the release of Cyto-c from mitochondria to cytoplasm, which further confirmed the initiation of apoptosis (Fig. 2d).

Above data elucidate that QIG triggers caspase-dependent apoptosis in CRC cells.

### Autophagy was significantly induced by QIG in CRC cells

**Fig. 3:**
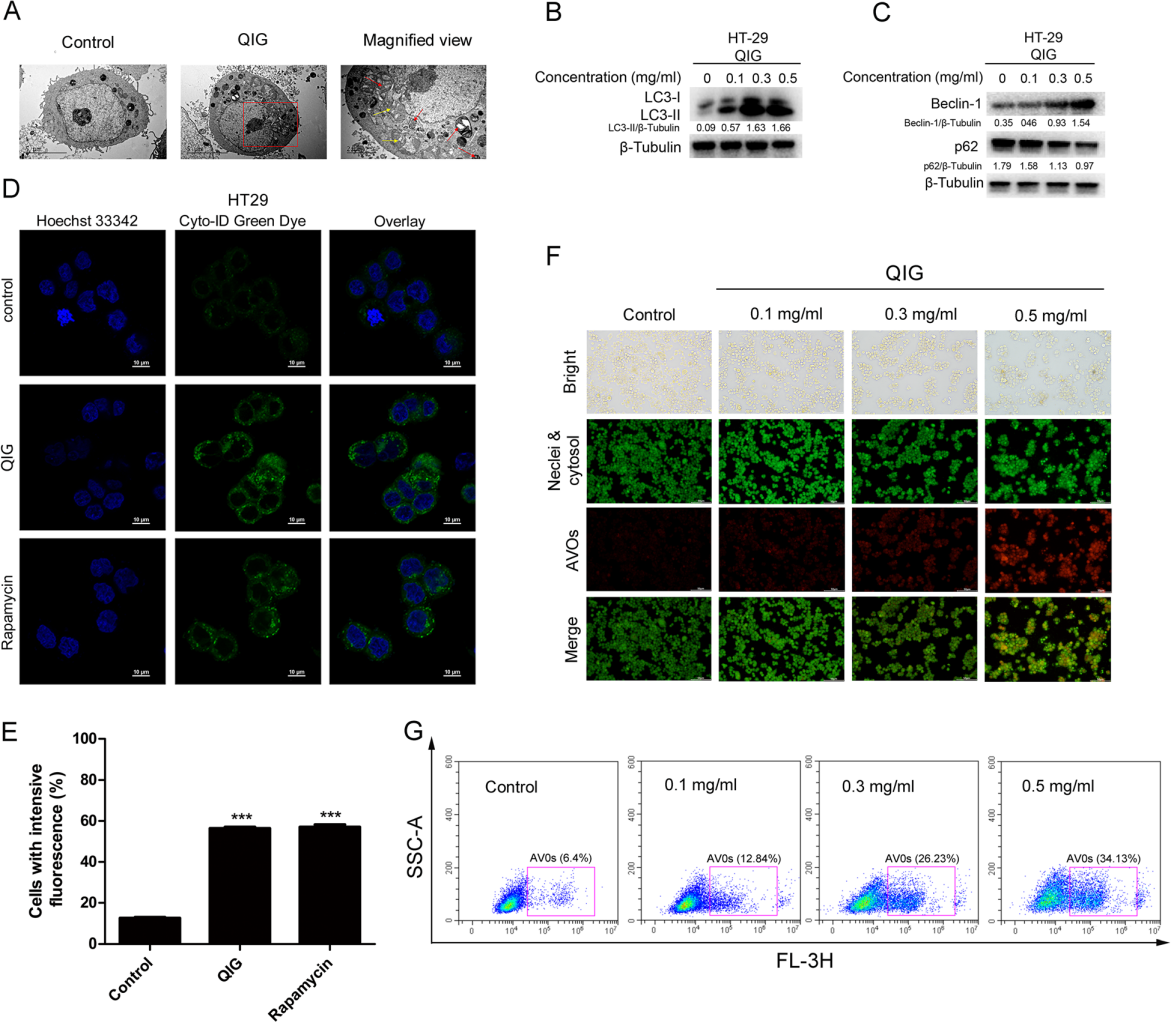
Autophagy was significantly induced by QIG in CRC cells. **a** After treatment with 0.3 mg/ml of QIG for 24 h, the ultrastructural analysis of HT-29 cells was assessed by TEM (yellow arrows indicated swollen mitochondria, red arrows indicated autophagosomes). **b**-**c** HT-29 cells were dose-dependently exposed to QIG, then the autophagy-related protein LC3-I/II, Beclin-1 and p62 were assessed by western blotting and quantified by Image J software. **d** HT-29 cells were administered with 0.3 mg/ml of QIG or positive control Rapamycin (50 nM) for 24 h and examined by confocal fluorescence microscopy after Cyto-ID® Green dye staining. **e** The quantitation analysis of (**d**) was performed by Image J software and the data were presented as mean ± SD (***P < 0.001). **f** The formation of AVOs was observed by fluorescence microscopy after AO staining in HT-29 cells treated with 0–0.5 mg/ml of QIG for 24 h. **g** The quantitation analysis of AVOs in (**f**) was performed by flow cytometry

We used several established methods to test whether autophagy was initiated by QIG in CRC cells. First, the formation and accumulation of autophagy vacuoles in HT-29 cells were analyzed by transmission electron microscopy (TEM) after administration with QIG for 24 h (Fig. 3a). We found that the autophagic vesicles in the administration group increased and the mitochondria were significantly swollen compared to the control. Moreover, western blotting was used to assess the level of autophagy-related proteins LC3-I/II, Beclin-1 and p62 [21]. Among them, LC3 was a marker protein of autophagy, Beclin-1 could regulate the formation of autophagosomes [22] and p62 was a selective substrate of autophagy [23]. As shown in Fig. 3b, endogenous LC3-II aggregated dose-dependently after treated HT-29 cells with QIG from 0 to 0.5 mg/ml, reflecting a significant change of LC3-I to LC3-II. At the same time, we found the expression of Beclin-1 was increased, and that of p62 was decreased as compared with the control group (Fig. 3c).

Next, Cyto-ID® Green was employed to detect autophagosomes in the QIG-treated HT-29 cells. Cells exposed to QIG showed autophagosomes with green fluorescence, similar to the cells administrated with rapamycin (positive control), while the blank control group did not have specific fluorescence (Fig. 3d and e). Finally, we further detected the formation of AVOs by fluorescence microscopy and quantified them by flow cytometry after AO staining in HT-29 cells treated with QIG from 0 to 0.5 mg/ml. Comparing with the control group, AVOs accumulated in a dose-dependent manner under a fluorescence view (Fig. 3f) and increased from 6.4 to 26.23% and 34.13% at 0.3 and 0.5 mg/ml, respectively (Fig. 3g).

Altogether, these data demonstrate that QIG induces autophagy in HT-29 cells.

### Inhibition of autophagy attenuated QIG-induced cytotoxicity in HT-29 cells

**Fig. 4:**
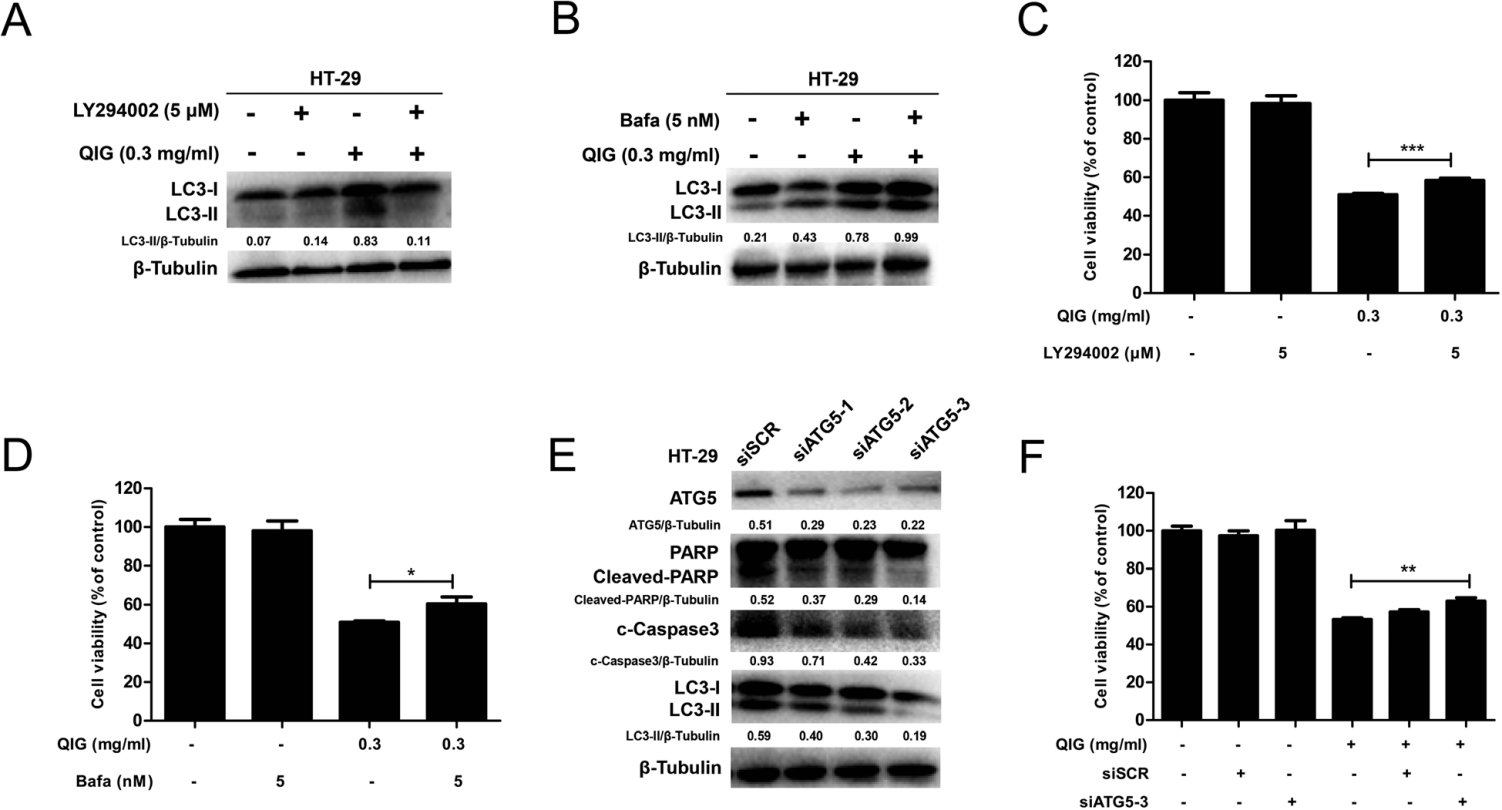
Inhibition of autophagy attenuated QIG-induced cytotoxicity in HT-29 cells. **a**-**b** Autophagy marker protein LC3-I/II were assessed by western blotting in HT-29 cells after treated with 0.3 mg/ml QIG in combination with or without 5 μM LY394002 or 5 nM BAF-1. **c**-**d** CCK8 assay was used to detect the cell viability (*P < 0.05, ***P < 0.001). **e** After cells were treated with QIG (0.3 mg/ml) and siATG5 for 24 h, the expression of proteins LC3-I/II and cleaved-PARP, cleave-caspase 3 were analyzed by western blotting and quantified by Image J software. **f** CCK8 assay was used to detect the cell viability (**P < 0.01)

Next, we used two autophagy inhibitors, Bafilomycin A-1 (BAF-1) and LY394002, to explore the function of autophagy in QIG’s cytotoxicity on CRC cells. We found that 5 μM of LY294002 significantly reduced the formation of autophagosomes as evidenced by the down-regulation of LC3-II, while BAF-1 at a concentration of 5 nM effectively blocked the binding of autophagosomes and lysosomes, leading to the up-regulation of LC3-II in HT29 cells (Fig. 4a and b). CCK8 test showed that the cytotoxicity induced by QIG was slightly attenuated after co-incubation with autophagy inhibitors (Fig. 4c and d). In addition, we also used siRNA to silence the autophagy-related gene ATG5. The western blotting demonstrated the level of autophagy marker protein LC3-II and apoptosis-related proteins was down-regulated in HT-29 cells after transfected with siRNA-ATG5 (Fig. 4e). At the same time, the cytotoxicity induced by QIG was decreased (Fig. 4f).

Taken together, we speculate that the autophagy triggered by QIG is non-protective in HT-29 cells.

### Intracellular ROS, Erk and AKT/mTOR signaling pathways were associated with the cytotoxicity triggered by QIG

**Fig. 5:**
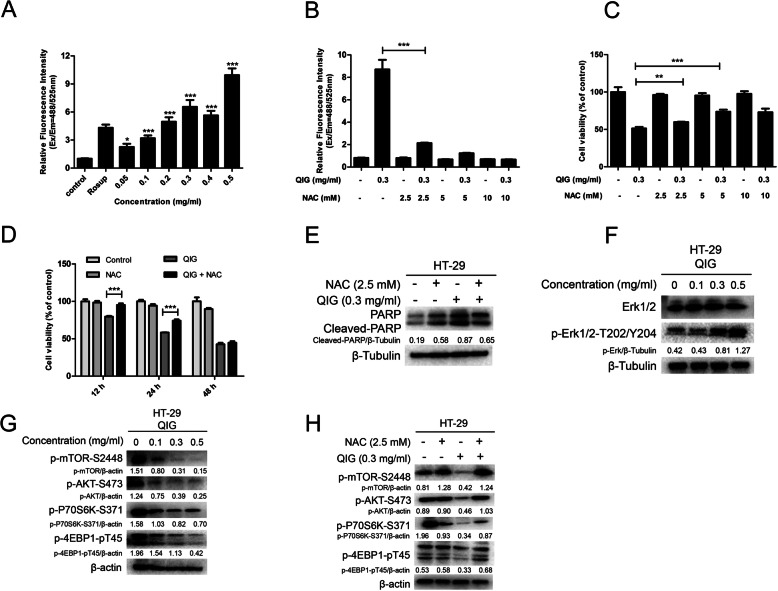
Intracellular ROS, AKT/mTOR and Erk signaling pathways were associated with the cytotoxicity induced by QIG. **a**-**b** Cells were treated with QIG in combination with or without NAC (2.5 mM, 5 mM and 10 mM) for 24 h. The level of intracellular ROS was assessed by fluorescence microplate reader after cells were treated with ROS assay kit (*P < 0.05, ***P < 0.001). **c**-**d** Cell viability was measured by CCK8 assay (**P < 0.01, ***P < 0.001). **e** Western blotting was used to detect the expression of protein cleaved-PARP and β-Tubulin. **f**-**g** HT-29 cells were dose dependently treated with QIG, then the protein levels of p-mTOR-S2448, p-AKT-S473, P-P70S6K-S371, p-4EBP1-pT45, Erk1/2, p-Erk1/2-T202/Y204, β-Tubulin and β-actin were assessed by western blotting and quantified by Image J software. **h** Cells were treated with QIG (0.3 mg/ml) in combination with or without 2.5 mM NAC for 24 h and the expression of proteins p-mTOR-S2448, p-AKT-S473, p-P70S6K-S371, p-4EBP1-pT45 and β-actin were analyzed by western blotting and quantified by Image J software

Many natural products provoked cytotoxicity on tumor cells through inducing the accumulation of intracellular reactive oxygen species (ROS) [24]. In this experiment, we detected the generation of ROS through Reactive Oxygen Assay kit after HT-29 cells were incubated with QIG at different concentrations for 24 h. The result showed that QIG significantly triggered the accumulation of intracellular ROS in a dose-dependent manner (Fig. 5a), and ROS inhibitor acetylcysteine (NAC) could obviously reduce the QIG-induced ROS at a concentration of 2.5 mM (Fig. 5b). The cytotoxicity triggered by QIG was remarkably reduced after scavenging ROS by NAC on HT-29 cells (Fig. 5c and d). Besides, the expression of apoptosis-associated protein cleaved-PARP in HT-29 cells induced by QIG was reduced after combined with ROS inhibitor (Fig. 5e).

It has been reported that the activated extracellular signal-regulated kinase (Erk1/2) could positively regulate the autophagy marker protein LC3, thereby promote the autophagic death of tumor cells [25]. Besides, AKT/mTOR was another important signaling pathway involved in both cell growth and autophagy. Studies have shown that intracellular ROS accumulation can suppress mTOR, then increase cell autophagy and growth inhibition [26, 27]. The results of western blotting experiments showed increased level of phosphorylated Erk1/2-T202/Y204, and decreased level of phosphorylated AKT, mTOR, 70S6K and 4EBP1 in HT-29 cells after treated with QIG (Fig. 5f and g). In addition, ROS inhibitor NAC could reverse the inhibition of mTOR signaling pathway by QIG (Fig. 5h).

Collectively, these results find that QIG triggers cytotoxicity on CRC cells through intracellular ROS accumulation, and both Erk and AKT/mTOR signaling pathway participate in the process of QIG-induced autophagic cell death.

### QIG could inhibit EMT process and migration of CRC cells

**Fig. 6:**
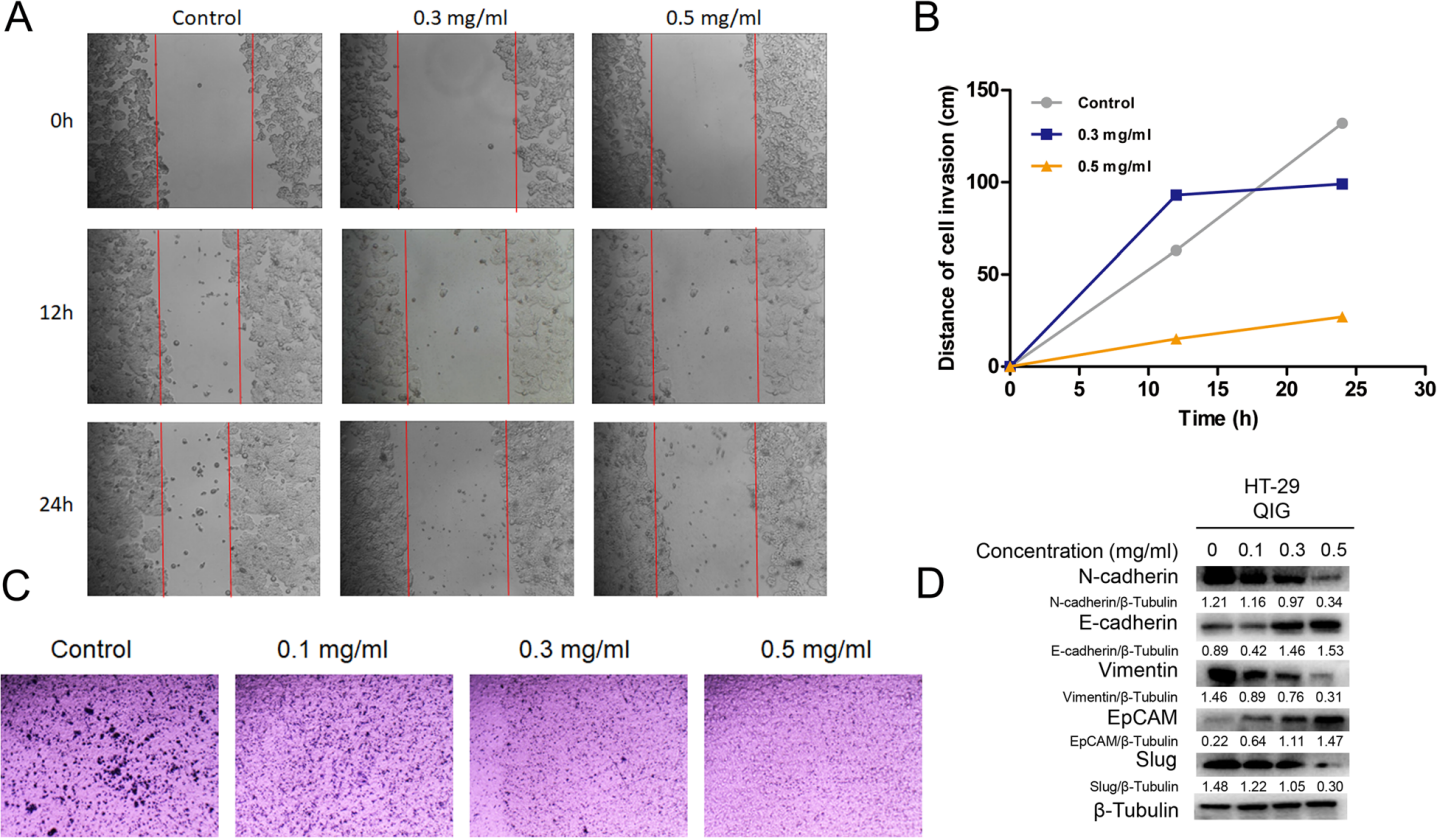
QIG could inhibit EMT process and migration of CRC cells. **a** After administration with 0.1 mg/ml, 0.3 mg/ml and 0.5 mg/ml of QIG, the migration of the cells was examined with an inverted microscope. **b** The migration distance of HT-29 cells were assessed by Excel 2019. **c** Transwell invasion assay was performed in HT-29 cells. **d** HT-29 cells were incubated with QIG 0.3 mg/ml for 24 h. The expression of N-cadherin, E-cadherin, vimentin, EpCAM, Slug and β-actin was assessed by western blotting and quantified by Image J software

Epithelial mesenchymal transition (EMT) participates in tumorigenesis, invasion and dissemination [28]. The wound healing assays indicated that the scratches of the control group were almost cured after 24 h, while the scratches of QIG-treated groups were sustained especially at the concentration of 0.5 mg/ml (Fig. 6a and b). In addition, transwell experiments were performed to further study the effects of QIG on cell migration. As shown in Fig. 6c, HT-29 cells of QIG-treated groups invaded significantly less than the control group. Furthermore, Fig. 6d showed increased level of EpCAM and E-cadherin, and decreased level of N-cadherin, Slug and Vimentin in HT-29 cells after treatment with QIG. In summary, QIG can significantly suppress EMT process and migration of CRC cells.

## Discussion

Despite current advances in cancer treatment strategies including targeted therapy and immunotherapy, the relatively low response rate, drug resistance, adverse effects as well as economic burden remain challenging for patient organism [29]. Natural materials, especially Chinese herbal medicine, have potential application as chemoprotective agents to decrease the risk of cancer. Natural products combined therapy may sensitize tumor to conventional chemotherapeutics and advanced immunotherapy through various mechanisms, thereby reducing the dosage of administered therapeutics and diminishing economic burden on the patients [30]. In addition, natural products are generally well tolerated even at high dosages. Therefore, natural products as well as their combination therapy represent a promising approach in the therapy of cancer. The propose of the present work is to dig the potential in cancer treatment of QIG water extract, the active constituent of Uyghur medicine KJA used for UC treatment, and we mainly focus on its direct cytotoxic effect on CRC cells.

First, through CCK8, flow cytometry and western blotting, we found that QIG extract could suppress the viability of CRC cells and trigger caspases-dependent apoptosis. Induction of apoptosis is an important mechanism for Chinese medicine to destroy cancer cells [31]. Bax, caspases, and PARP protein family play key roles in the process of apoptosis, and they have complex relationship with each other. The Cyto-c can be released by pro-apoptotic effector Bax into cytoplasm via mitochondrial pathway [32], then causing the cleavage of caspase-3 and caspase-9 [33]. The cascade-like cleavage of caspases can further catalyze the hydrolysis of its downstream protein PARP, which ultimately results in cell apoptosis. In this study, the increased level of Bax and the cleavage of caspase-3, caspase-9, PARP, as well as the down-regulated Cyto-c in the mitochondria and up-regulated Cyto-c in the cytoplasm in HT-29 cells treated with QIG suggested that caspase-dependent apoptosis was induced by QIG extract.

Subsequently, we proved for the first time that QIG extract also triggered autophagy in CRC cells as evidenced by the appearance of autophagosomes, increased expression level of LC3-II and Beclin-1, decreased expression level of p62 and the production of LC3-positive autophagic-like vacuoles detected by TEM, western blotting and confocal microscope respectively. The formation of AVOs trigged by QIG were detected through fluorescence microscopy and quantified by flow cytometry. Autophagy can play contradictory roles in the development of tumor. According to different tumor types and circumstances, it can act as tumor inhibitor or assist cancer cells to survive the metabolic stress and the cytotoxicity of therapeutic drugs [34]. To investigate whether autophagy induced by QIG enhances or attenuates cytotoxicity, we used two autophagy inhibitors, BAF-1 and LY294002. BAF-1 impedes autophagy by preventing the fusion of lysosome and autophagy, while LY294002 blocks the formation of autophagosomes. CCK8 and western blotting test showed that the cytotoxicity induced by QIG was not enhanced but slightly attenuated after co-incubation with these two autophagy inhibitors. Similar conclusions were drawn from the siRNA interference test. Taken together, we speculated that QIG induced autophagic cell death (ACD) in CRC HT-29 cells.

We further explored the possible regulatory signals involved in the QIG-triggered cytotoxicity on CRC cells. Many lines of evidence suggest that ROS may act as a second messenger to upregulate the expression of pro-apoptotic proteins, activate caspases and ultimately induce apoptosis. In this study, we found that QIG could increase ROS accumulation in HT-29 cells and antioxidant NAC could partially reverse the cytotoxicity and apoptosis induced by QIG, suggesting that ROS was one of the regulators participating in the QIG-induced cytotoxicity. The Akt/mTOR signaling pathway was a classical negative regulator of autophagy, which positively regulated protein translation by the phosphorylation of AKT, mTOR and its downstream substance p70S6K and 4EBP1 [35]. Intracellular ROS is also the main signal mediator that maintains autophagy which inhibits the AKT/mTOR signaling pathway through PAPR-1-LKBI-AMPK and P13K/AKT pathways, finally inducing cell autophagy [36]. The Erk signal is another major pathway regulating autophagy in eukaryotic cells, and studies have confirmed that activated Erk signaling pathway can promote tumor cell apoptosis and autophagic cell death [26, 37]. Our study demonstrated that after treatment with QIG, the phosphorylation levels of AKT, mTOR and two downstream proteins (p70S6K and 4EBP1) decreased in HT29 cells, while Erk phosphorylation increased. The above experiments proved Erk and AKT/mTOR signaling pathways participated in tumor autophagic cell death induced by QIG. In addition, the combined use of ROS inhibitor NAC could reverse the inhibition of AKT/mTOR signaling pathway by QIG, suggesting that the QIG-induced ROS accumulation also triggered autophagic cell death and cell growth inhibition by suppressing the AKT/mTOR pathway.

Considering the importance of metastasis in tumor development, we also investigated the effect of QIG extract on CRC cell EMT, a significant symbol of tumor migration and invasion [28]. Loss of epithelial cell phenotype and acquisition of interstitial properties are the main features of EMT. In our work, western blotting tests indicated the level of E-cadherin and EpCAM in HT-29 cells was up-regulated after the treatment of QIG, while the mesenchymal marker proteins such as N-cadherin, vimentin and Slug were remarkably down-regulated. At the same time, wound healing and invasion tests also demonstrated that QIG could inhibit migration of CRC cells.

## Conclusion

**Fig. 7:**
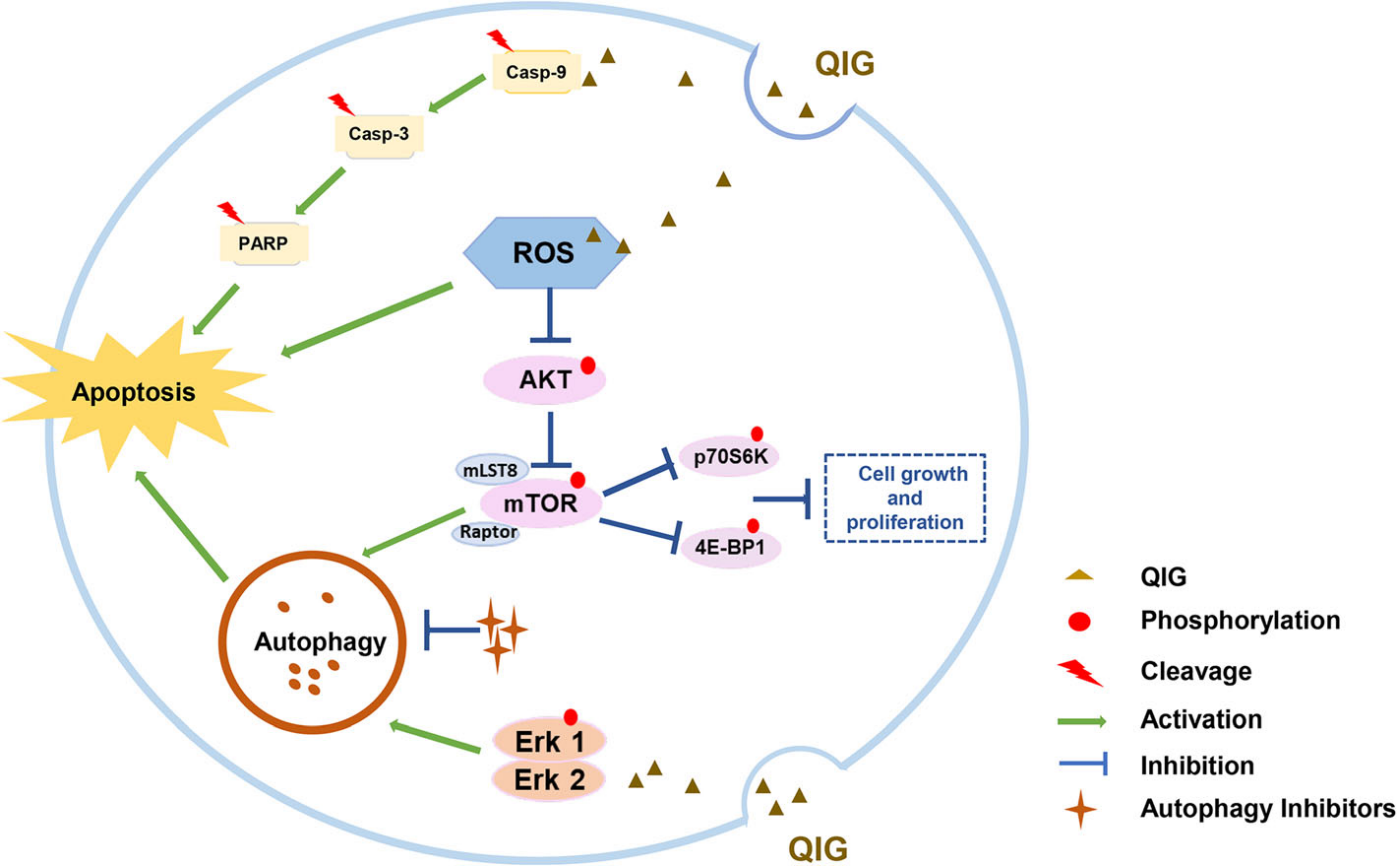
Overview of Uyghur medicine QIG triggers autophagy and apoptosis in colorectal cancer cells

In summary, our research demonstrates that QIG can simultaneously trigger caspase-dependent apoptosis and autophagic cell death which together contribute to its cytotoxic effect on CRC cells, and this cytotoxicity is related to the intracellular ROS accumulation which can suppress the AKT/mTOR signaling pathway. Additionally, Erk signaling pathways are also involved in the process of autophagic cell death. Moreover, QIG can affect the EMT process and inhibit CRC cell migration (Fig. 7). Therefore, as a Uyghur medicine for UC treatment, QIG has a potential to be used in CRC therapy as a complement and alternative medicine. However, for the effective application of QIG in CRC prevention and treatment, the main active ingredients in QIG water extract as well as the in vivo anti-tumor activity should be investigated in the following study. The present data provide experimental basis for these further explorations.

## Data Availability

The datasets for supporting the outcomes of the study are included in the article and additional information can be provided on request made to the corresponding author.

## Abbreviations

CRC: Colorectal Cancer
QIG: Quercuse infectoria galls
TEM: Transmission Electron Microscopy
ROS: Reactive Oxygen Species
UC: Ulcerative Colitis
ACD: Autophagic Cell Death
NAC: N-acetylcysteine amide
Bafa: Bafilomycin A1
